# Development of admixture mapping panels for African Americans from commercial high-density SNP arrays

**DOI:** 10.1186/1471-2164-11-417

**Published:** 2010-07-05

**Authors:** Guanjie Chen, Daniel Shriner, Jie Zhou, Ayo Doumatey, Hanxia Huang, Norman P Gerry, Alan Herbert, Michael F Christman, Yuanxiu Chen, Georgia M Dunston, Mezbah U Faruque, Charles N Rotimi, Adebowale Adeyemo

**Affiliations:** 1 Center for Research on Genomics and Global Health, National Human Genome Research Institute, National Institutes of Health, Bethesda, Maryland 20892 USA; 2 Coriell Institute for Medical Research, Camden, NJ 08103 USA; 3 Department of Genetics and Genomics, Boston University School of Medicine, Boston, Massachusetts 02118 USA; 4 National Human Genome Center, Howard University, Washington DC 20060 USA

## Abstract

**Background:**

Admixture mapping is a powerful approach for identifying genetic variants involved in human disease that exploits the unique genomic structure in recently admixed populations. To use existing published panels of ancestry-informative markers (AIMs) for admixture mapping, markers have to be genotyped *de novo *for each admixed study sample and samples representing the ancestral parental populations. The increased availability of dense marker data on commercial chips has made it feasible to develop panels wherein the markers need not be predetermined.

**Results:**

We developed two panels of AIMs (~2,000 markers each) based on the Affymetrix Genome-Wide Human SNP Array 6.0 for admixture mapping with African American samples. These two AIM panels had good map power that was higher than that of a denser panel of ~20,000 random markers as well as other published panels of AIMs. As a test case, we applied the panels in an admixture mapping study of hypertension in African Americans in the Washington, D.C. metropolitan area.

**Conclusions:**

Developing marker panels for admixture mapping from existing genome-wide genotype data offers two major advantages: (1) no *de novo *genotyping needs to be done, thereby saving costs, and (2) markers can be filtered for various quality measures and replacement markers (to minimize gaps) can be selected at no additional cost. Panels of carefully selected AIMs have two major advantages over panels of random markers: (1) the map power from sparser panels of AIMs is higher than that of ~10-fold denser panels of random markers, and (2) clusters can be labeled based on information from the parental populations. With current technology, chip-based genome-wide genotyping is less expensive than genotyping ~20,000 random markers. The major advantage of using random markers is the absence of ascertainment effects resulting from the process of selecting markers. The ability to develop marker panels informative for ancestry from SNP chip genotype data provides a fresh opportunity to conduct admixture mapping for disease genes in admixed populations when genome-wide association data exist or are planned.

## Background

Admixture mapping is an approach for localizing disease susceptibility loci that attempts to capitalize on the long-range linkage disequilibrium occurring in populations formed by recent mixing of ancestral populations [1-6]. The approach uses samples from recently admixed populations to detect susceptibility loci at which the risk alleles have different frequencies in the ancestral parental populations. Admixture mapping is an economical and theoretically powerful approach. Compared to linkage, admixture mapping does not require families and has more power. Compared to association, admixture mapping requires ~200-500-fold fewer markers, is not susceptible to allelic heterogeneity, and can be used with either case-only or case-control study designs. Admixture mapping can also be performed with generalized linear models to accommodate quantitative traits [1]. Admixture mapping has been performed for many complex traits which exhibit strong differences in prevalence across ethnicities, such as end-stage renal disease [7,8], hypertension [9-11], multiple sclerosis [12], obesity [13-15], peripheral arterial disease [16], prostate cancer [17,18], rheumatoid arthritis [19], serum inflammatory markers [20], systemic lupus erythematosus [21], type 2 diabetes [22], and white blood cell count [23].

Several groups have built panels of ancestry-informative markers (AIMs) based on multiple databases of human genetic variation [24-27]. Previously, admixture mapping required the construction of panels of AIMs based on screening large reference sets of genetic variation for ancestry-informative markers followed by *de novo *genotyping at those preselected markers in the admixed study sample and the samples representing the (putative) ancestral parental populations [12,20,23,26,28,29]. However, given commercially available high-density marker arrays, it is now possible to construct customized panels from markers already genotyped in the admixed study sample(s) [27,30-34].

In this study, we constructed marker panels for admixture mapping with African American populations, starting from the Affymetrix Genome-Wide Human SNP Array 6.0, which probes variation at 909,508 single-nucleotide polymorphisms (SNPs). Using genome-wide genotypes in our study sample of African Americans already experimentally determined for genome-wide association studies and HapMap data to represent the presumed ancestral parental populations, we constructed one panel consisting of SNPs with large differences in allele frequencies between the ancestral parental populations and a second panel consisting of SNPs with large *F_ST _*values between the ancestral parental populations. We also constructed a panel consisting of random markers not selected to be ancestrally informative. Characteristics of these panels, including the number of markers and information content, are presented. As a test case, we apply these panels to a study of hypertension in African Americans.

## Methods

### Study Population

The admixed population under study comprised participants in the Howard University Family Study (HUFS) from the Washington, D.C. metropolitan area [35]. The first phase of recruitment involved enrolling and examining a randomly ascertained cohort of African American families with members in multiple generations. To facilitate nested case-control study designs, additional unrelated individuals from the same geographic area were enrolled in a second phase of recruitment. Participants were not ascertained based on any phenotypes. Participants were interviewed and measured for various anthropometric and clinical variables. Blood pressure was measured in the sitting position using an oscillometric device (Omron Healthcare, Kyoto, Japan). Three readings were taken with a ten minute interval between readings. The reported systolic and diastolic blood pressure readings were the average of the second and third readings. Hypertension case status was defined as systolic blood pressure ≥ 140 mmHg, or diastolic blood pressure ≥ 90 mmHg, or treatment with antihypertensive medication. We identified a subset of 1,017 unrelated individuals including 509 hypertensive cases and 508 controls for use in admixture mapping.

Genome-wide genotyping in the HUFS was performed using the Affymetrix Genome-Wide Human SNP Array 6.0. DNA samples were prepared and hybridized following the manufacturer's instructions [35]. Genotype calls were made using the Birdseed algorithm, version 2 [36]. We had four inclusion criteria: the individual sample call rate had to be ≥ 95% (no samples excluded), the SNP call rate had to be ≥ 95% (41,885 SNPs excluded), the minor allele frequency had to be ≥ 0.01 (19,154 SNPs excluded), and the *p*-value for the Hardy-Weinberg (HWE) test of equilibrium had to be ≥ 1.0×10^-3 ^(6,317 SNPs excluded). After filtering, 842,074 autosomal and X chromosomal SNPs remained.

HapMap phase III CEU (1,403,896 SNPs and 180 individuals), YRI (1,484,416 SNPs and 180 individuals), and ASW (1,536,247 SNPs and 90 individuals) genotype data were obtained from the International HapMap Project http://hapmap.ncbi.nlm.nih.gov/downloads/genotypes/2008-07_phaseIII/. We retained unrelated individuals, leaving 109 CEU individuals, 108 YRI individuals, and 55 ASW individuals. We used the same criteria (sample call rate > 95%, locus call rate ≥ 95%, minor allele frequency > 0.01, HWE *p *≥ 1.0×10^-3^) for filtering genotypes. After filtering, the intersection of the CEU, YRI, and HUFS data sets included 708,383 SNPs. We used these contemporary samples of 109 unrelated CEU individuals and 108 unrelated YRI individuals as proxy samples for the presumed ancestral parental populations of our African American sample.

### *δ *and *F_ST _*Calculations

For a given SNP, *δ *was calculated as the absolute difference in allele frequencies in the CEU and YRI data, *δ *= |*p*_*CEU *_- *p*_*YRI*_|. Wright [37] suggested the fixation index *F_ST _*to evaluate population differentiation. We estimated *F_ST _*between the CEU and YRI samples using the formula  in which  and . Wright [38] suggested qualitative guidelines for the interpretation of *F_ST_*: values from 0 to 0.05 indicate little population differentiation, values between 0.05 and 0.15 indicate moderate population differentiation, values between 0.15 and 0.25 indicate large population differentiation, and values above 0.25 indicate very large population differentiation.

### Genetic Map of SNPs

The Rutgers Combined Linkage-Physical Map of the Human Genome was used to locate markers on the genetic map (in cM) given positions on the physical map (in bp). The positions of SNPs on the genetic map were obtained using a web-based application http://integrin.ucd.ie/cgi-bin/rs2cm.cgi.

### Selection of Ancestry-Informative Markers from HapMap Data

We followed a six-step process to select AIMs. First, we selected SNPs for which the minor allele frequency was ≥ 0.01 in both ancestry populations (CEU and YRI). Second, we filtered for SNPs for which *δ *≥ 0.6 between CEU and YRI. Third, we divided each chromosome into consecutive, non-overlapping bins of size 1 Mb and sorted the SNPs within each bin in descending order according to the *δ *values. Fourth, for each chromosome, we estimated pairwise correlations between the top-ranked SNPs across the bins. Fifth, for each pair of SNPs, if *r*^2 ^≥ 0.4 in either the CEU or YRI sample, we discarded the SNP with the smaller *δ *value from its bin and promoted all remaining SNPs in that bin. If *δ *values were equal (to the fourth decimal place), we discarded the distal SNP. We iterated steps 4-5 until *r*^2 ^< 0.4 in either of the CEU or YRI sample for all pairs of top-ranked SNPs per bin. The resulting panel comprised 2,076 AIMs. We repeated this entire process based on *F_ST _*≥ 0.4, yielding a second panel consisting of 1,923 AIMs. Given *δ *= 0.6, the allowable values of *F_ST _*range from *δ*^2 ^= 0.36 to [39]. Similarly, given *F_ST _*= 0.4, the allowable values of *δ *range from  to [39]. These calculations show the comparability of the two thresholds.

### Information Content and Map Power

We calculated the Shannon information content (SIC), defined as

in which *a*_00 _= (1 - *m*) × *p_YRI_*, *a*_01 _= *m *× *p_CEU_*, *a*_10 _= (1 - *m*) × (1 - *p_YRI_*), *a*_11 _= *m *× (1 - *p_CEU_*), and *m *is the proportion of European ancestry.

For a locus *i *and individual *j, X_ij _*was defined as the entropy of the locus-specific ancestry estimate and *G_j _*was defined as the entropy of the genome-wide ancestry estimate. The relative power at locus *i *was defined as . If *X_ij _*= *Gj *for all *j*, then *r_i _*= 0 and there is no additional information about local ancestry beyond information about genome-wide ancestry. If *X_ij _*= 0 for all *j*, then *r_j _*= 1 and there is perfect information for local ancestry [31]. The statistic *r*_*i *_and the average of *r_i _*across loci, *r_avg_*, were estimated using ANCESTRYMAP [3]. Relative to a study with perfect information about local ancestry (*r_avg _*= 1), 1/*r_avg _*times as many samples must be genotyped to achieve comparable power [31].

### Estimation of Individual Admixture and Population Structure

We used the variance inflation factor (VIF) to prune markers in linkage disequilibrium (LD). The VIF is equal to,  in which  is the multiple correlation coefficient. A VIF of 1 implies that the index SNP is completely independent of all other SNPs. Starting from a common set of SNPs passing quality control among the HapMap CEU, HapMap YRI, and HUFS data sets, we used LD-based pruning (VIF 1.1, window size 50 SNPs, window slide of 5 SNPs) to generate a set of 74,546 SNPs with minimal LD between the markers. We then randomly selected one-third of the SNPs to obtain a random marker panel (21 k random panel) that had 10-fold greater marker density than the AIMs panels. We also generated an additional panel (2 k random panel) by randomly sub-sampling 10% of the 21 k random panel to match the marker density of the AIMs panels. We examined clustering using a parametric approach implemented in STRUCTURE [40] and a nonparametric approach implemented in AWclust [41]. Analysis was performed in STRUCTURE without any prior population assignment and was performed ten times for each number of clusters (*K*), with 10,000 burn-in steps and a run length of 10,000 steps under the admixture model. We recorded the log likelihood of each analysis conditional on *K *estimated by STRUCTURE. Compared with this parametric approach, the nonparametric approach in AWclust [41] uses allele-sharing distance (ASD) and Ward's minimum variance algorithm to cluster the individuals in the ASD matrix. AWclust does not assume Hardy-Weinberg equilibrium or linkage equilibrium and does not require allele frequency estimates. We varied *K *from one to six in both programs.

### Application of the panels to a study of hypertension

Two statistics were used to test for the presence of disease loci using ANCESTYMAP [3]. One was the locus-genome statistic, which compared the admixture proportion at one locus with the genome-wide average among cases only. The locus-genome statistic was tested via a likelihood-ratio statistic, *i.e*., the likelihood of a locus being a disease locus to the likelihood of the locus not being a disease locus. The *LOD *score was defined as the likelihood-ratio test statistic divided by 2ln(10). The genome-wide significance threshold of the *LOD *score was set at 2 [3]. The other statistic was the case-control statistic, which compared cases with controls at every point in the genome, testing for differences in ancestry estimates. A deviation from the genome-wide average of one parental population ancestry seen in cases but not in controls provided evidence of a disease locus. The case-control statistic followed the standard normal distribution under the null hypothesis that a locus was not a disease locus. The genome-wide significance threshold of the *z*-statistic was set at ± 4.2 for the two panels of AIMs and ± 4.7 for the panel based on random markers. We specified in the disease model that the relative risk for hypertensive heart disease among African Americans was 2.80 compared to European Americans [26].

## Results

### Marker Panels for Admixture Mapping in African Americans

**Figure 1:**
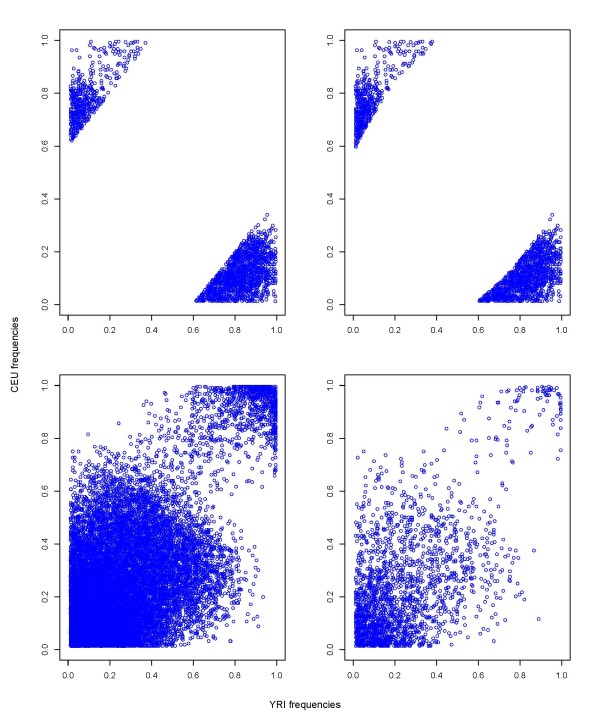
**HapMap phase III CEU and YRI allele frequencies**. Top left) Panel based on *δ*. Top right) Panel based on *F_ST_*. Bottom left) Panel based on 21 k random markers. Bottom right) Panel based on 2 k random markers.

**Figure 2:**
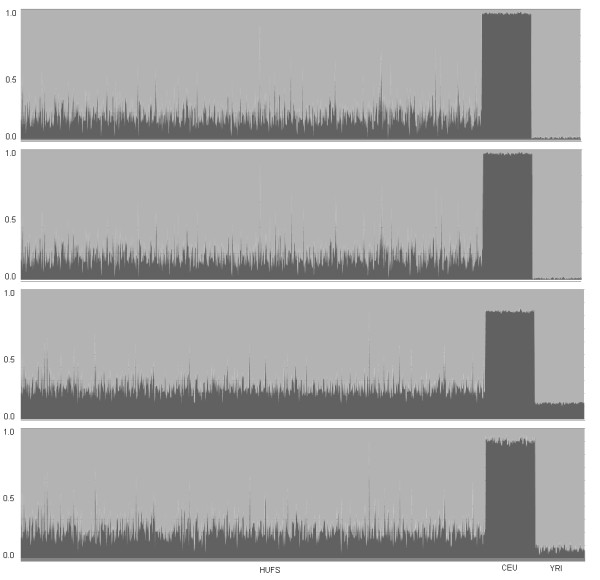
**Summary plots from STRUCTURE analysis of individual admixture proportions in HUFS, CEU, and YRI, conditional on *K *= 2 populations**. From top to bottom, admixture proportions using the panels based on *δ*, *F_ST_*, 2 k random markers, and 21 k random markers.

The distribution of SNPs across the AIMs panels (one based on *δ *contained 2,076 AIMs (Additional file 1), the other based on *F_ST _*contained 1,923 AIMs (Additional file 2)) and two random marker panels (21 k random marker panel and 2 k random marker panel, Additional file 3) are shown in Table 1. The panels covered all 22 autosomes and the X chromosome (Table 1). All marker panels showed lower heterozygosities in the parental samples than in the admixed sample, with the two panels of AIMs showing ascertainment effects of lower heterozygosities in the parental samples and higher heterozygosity in the admixed sample (Table 2). Scatter plots of allele frequencies for AIMs showed clear differentiation of the two parental populations (Figure 1), as did the STRUCTURE plot assuming *K *= 2 populations (Figure 2) and the AWclust plot (Additional file 4). Excluding centromeres, the average inter-marker distance was 1.33 cM for the panel based on *δ*, 1.43 cM for the panel based on *F_ST_*, 0.124 cM for the panel based on 21 k random markers, and 1.17 cM for the panel based on 2 k random markers (Additional file 5). The average values of *δ*, *F_ST_*, and SIC were 0.715, 0.519, and 0.300 for the *δ *panel, 0.708, 0.531, and 0.308 for the *F_ST _*panel, 0.142, 0.049, and 0.026 for the 21 k random marker panel, and 0.143, 0.050, and 0.026 for the 2 k random marker panel, respectively (Additional file 6).

**Table 1:** Distribution of markers

	HapMap SNPs included in GWAS data	Number of markers (start position - end position in Mb)			
Chromosome		Panel based on *δ*	Panel based on *F_ST_*	Panel based on 21 k random markers	Panel based on 2 k random markers
1	55,147	167 (0.8-247.2)	150 (0.8-247.2)	1,734 (0.7-247.1)	171 (2.3-245.7)
2	58,091	184 (0.6-241.7)	177 (0.6-241.7)	1,545 (0.4-242.7)	155 (0.6-241.0)
3	47,963	139 (0.3-199.1)	132 (0.3-199.1)	1,376 (0.3-198.9)	137 (0.4-198.2)
4	43,771	139 (2.2-190.9)	131 (2.2-190.9)	1,327 (0.3-191.0)	143 (0.7-187.2)
5	44,805	115 (1.0-180.6)	106 (1.0-180.6)	1,303 (0.5-180.6)	133 (0.5-180.4)
6	44,882	107 (0.9-170.4)	95 (0.9-170.4)	1,182 (0.1-170.8)	146 (0.2-170.8)
7	37,440	112 (1.2-158.5)	104 (1.2-158.5)	1,126 (0.2-158.5)	98 (1.3-157.5)
8	38,149	115 (0.3-144.9)	107 (0.3-144.9)	1,006 (0.4-146.1)	97 (1.1-143.1)
9	32,770	82 (0.3-140.1)	76 (0.3-140.1)	1,003 (0.2-140.2)	91 (0.9-140.2)
10	37,715	105 (0.7-135.2)	98 (0.7-135.2)	1,042 (0.7-135.3)	107 (1.3-130.6)
11	34,812	92 (2.6-132.9)	84 (2.6-132.9)	965 (0.2-134.5)	93 (1.9-134.2)
12	33,366	87 (0.3-131.1)	75 (0.3-131.1)	1,072 (0.1-132.1)	101 (1.8-129.5)
13	26,886	63 (18.9-113.8)	61 (18.9-113.8)	824 (18.2-114.1)	87 (18.2-112.3)
14	22,056	68 (19.9-106.3)	61 (19.9-106.3)	742 (19.5-105.1)	70 (19.5-102.9)
15	20,351	66 (20.6-99.6)	59 (20.6-99.6)	726 (20.3-99.9)	69 (20.4-99.2)
16	21,527	60 (0.9-88.7)	58 (0.9-86.1)	776 (0.1-88.6)	66 (3.0-87.6)
17	16,077	53 (0.1-74.5)	49 (0.1-74.5)	708 (0.1-78.6)	76 (0.8-76.0)
18	20,776	49 (1.0-75.7)	46 (1.0-75.7)	722 (0.3-76.1)	72 (0.3-75.5)
19	9,560	38 (1.6-62.2)	36 (1.6-63.0)	488 (0.3-63.7)	42 (1.0-63.7)
20	18,133	52 (0.9-61.2)	50 (0.9-61.2)	648 (0.0-62.4)	60 (0.3-61.5)
21	9,948	26 (15.9-45.3)	24 (15.9-45.3)	344 (13.3-46.8)	40 (14.8-46.7)
22	8,831	24 (17.0-48.7)	21 (17.0-48.7)	415 (15.3-49.5)	46 (15.3-49.5)
X	25,327	133 (0.8-154.1)	123 (2.6-154.1)	563 (0.1-154.7)	69 (0.2-152.6)
Total	708,383	2,076	1,923	21,637	2,169

**Table 2:** Average heterozygosities

	Panel based on *δ*	Panel based on *F_ST_*	Panel based on 21 k random markers	Panel based on 2 k random markers
	
Sample	Observed Heterozygosity (Expected Heterozygosity)	Observed Heterozygosity (Expected Heterozygosity)	Observed Heterozygosity (Expected Heterozygosity)	Observed Heterozygosity (Expected Heterozygosity)
CEU	0.242 (0.240)	0.230 (0.229)	0.278 (0.276)	0.278 (0.277)
HUFS	0.385 (0.389)	0.383 (0.387)	0.281 (0.280)	0.283 (0.283)
YRI	0.229 (0.228)	0.226 (0.225)	0.267 (0.266)	0.271 (0.269)

The two panels of AIMs shared 1,745 markers. The remaining markers (331 in the panel based on *δ*, 178 in the panel based on *F_ST_*) showed no significant difference in Shannon information content (*SIC*) (*t*-test, *p *= 0.10). The *δ *and *F_ST _*values in the two panels were highly positively correlated (*r *= 0.92, *p *< 0.0001). The *δ *in the panel based on *δ *was significantly higher than *δ *estimated from the panel based on *F_ST _*(*p *= 0.0004). Similarly, *F_ST _*in the panel based on *F_ST _*was significantly higher than *F_ST _*in the panel based on *δ *(*p *< 0.0001).

### Sample Characteristics

The genome-wide average *F_ST _*between HUFS and YRI was 0.0295, indicating little population differentiation. The genome-wide average *F_ST _*was 0.0656 between HUFS and CEU and 0.0753 between CEU and YRI, both indicating moderate population differentiation. As expected, these results indicated that our admixed HUFS sample was more similar to YRI than CEU, *i.e*., the proportion of African ancestry exceeded the proportion of European ancestry. Similarly, principal coordinate analysis showed that the HUFS sample was intermediate between the two ancestral parental populations and on average closer to YRI than CEU (Additional file 4). The estimated proportions of African ancestry in the HUFS sample using ANCESTRYMAP were 0.81 ± 0.11 and 0.84 ± 0.08 for the autosomes and the X chromosome, respectively.

### Admixture Information Content

**Figure 3:**
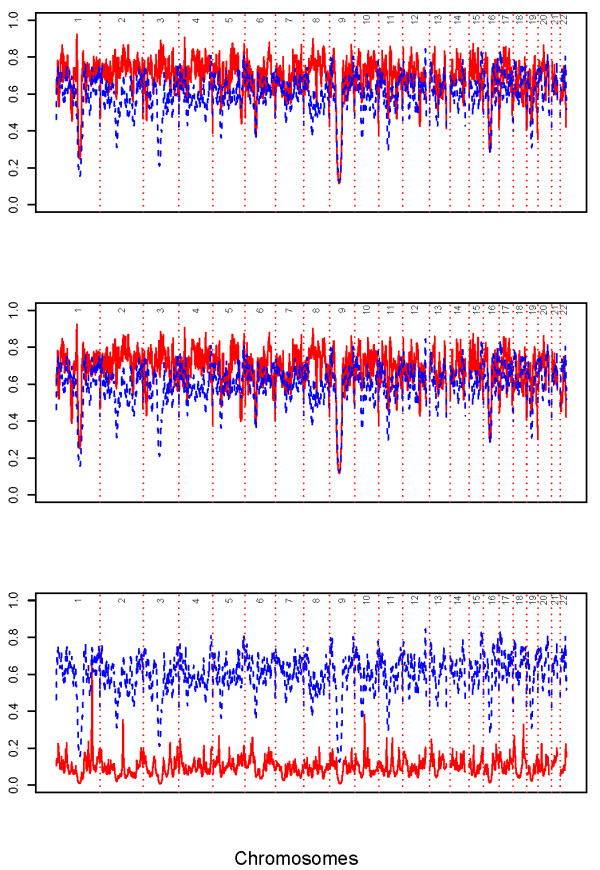
**Map power**. Blue represents *r_i _*for the panel of 21 k random markers (*r_avg _*= 0.65) and red represents *r_i _*for the compared panel. Top) Panel based on *δ *(*r_avg _*= 0.73). Middle) Panel based on *F_ST _*(*r_avg _*= 0.73). Bottom) Panel based on 2 k random markers (*r_avg _*= 0.13).

**Figure 4:**
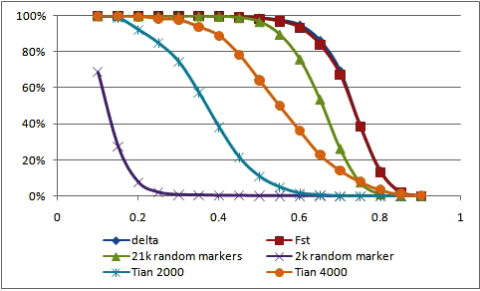
**Percentage of markers with threshold values of *r_i_***.

We evaluated the informativeness of the two panels of random markers compared to the informativeness of the two panels of AIMs. The proportions of markers in the panel of 21 k random markers for which *r_i _*≥ 0.50, *r_i _*≥ 0.75, and *r_i _*≥ 0.80 were 96.74%, 7.68%, and 1.20%, respectively, and the panel had a map power of *r_avg _*= 0.65. The proportions of markers in the panel of AIMs based on *δ *for which *r_i _*≥ 0.50, *r_i _*≥ 0.75, and *r_i _*≥ 0.80 were 98.82%, 38.86%, and 2.28%, respectively. The panel of AIMs based on *F_ST _*yielded values similar to those from the panel of AIMs based on *δ *values. The map power was *r_avg _*= 0.73 for the panels based on *δ *and *F_ST _*(Figures 3 and 4). The proportion of markers in the panel based on 2 k random markers for which *r_i _*≥ 0.50, *r_i _*≥ 0.75, and *r_i _*≥ 0.80 were 0.19%, 0%, and 0%, respectively, and the panel had a map power of *r_avg _*= 0.13 (Figures 3 and 4). These estimates indicate that the two panels of AIMs extracted more ancestry information than a 10-fold denser panel of random markers and much more than the 2 k random marker panel. Using the *r_avg _*statistic, one would need to study 1.37 (= 1/0.73), 1.37 (= 1/0.73), 1.54 (= 1/0.65), and 7.69 (= 1/0.13) times as many samples to maintain power to detect disease genes as would be necessary if one had full ancestry information, using the panels based on *δ*, *F_ST_*, 21 k random markers, and 2 k random markers, respectively.

We constructed panels conditional on approximate linkage equilibrium over 1 Mb bins. Our iterative pruning procedure was designed to avoid gaps in coverage and to eliminate background linkage disequilibrium. To compare our panels with previously published panels, we obtained two panels of AIMs developed for African Americans by Tian *et al*. [28]. From their panel of 4,222 AIMs, 682 AIMs were in common with the CEU, YRI, and HUFS data sets and all 682 AIMs passed quality control. Similarly, 321 AIMs from their panel of 2,000 AIMs were in common with the CEU, YRI, and HUFS data sets and all 321 AIMs passed quality control. As a result of the substantial reduction in marker density, the map power was reduced for both panels of Tian *et al*. using our HUFS data set (Table 3). The substantial reduction in marker density occurred because the panels of Tian *et al*. were developed independently of the Affymetrix chip we used for genotyping our sample and there was little overlap in the SNPs in their panels and on the chip. To investigate if this limitation also applied to another African American data set, we obtained the HapMap phase III ASW data. In the ASW data set, ~50% of the AIMs in either panel of Tian *et al*. were present, compared to > 98% of the AIMs from our panels, whereas almost every AIM present in the data passed quality control (Table 4). These comparisons highlight the advantage of being able to customize a panel using preexisting GWAS genotypes, especially for filling in gaps to improve coverage.

**Table 3:** Comparison of map power for different panels using HUFS

Panel (# of AIMs)	# of AIMs used (% Passed QC) ^1^	Map power ^2^	Reference
Based on *δ *(2,076)	1,943 (100%)	0.73	This manuscript
Based on *F_ST _*(1,923)	1,800 (100%)	0.73	This manuscript
21 k random markers (21,637)	21,074 (100%)	0.65	This manuscript
2 k random markers (2,169)	2,100 (100%)	0.13	This manuscript
Tian 2000 (2,000)	321 (100%)	0.37	[28]
Tian 4222 (4,222)	682 (100%)	0.56	[28]

^1 ^We compared the panels using all autosomal AIMs with quality control criteria locus call rate ≥ 95%, minor allele frequency > 0.01, and HWE *p *≥ 1.0×10^-3^.

^2 ^Map power (*r_avg_*) based on 1,017 individuals in the HUFS data set.

**Table 4:** Percentages of markers passing quality control for different panels using the HapMap ASW sample

Panel (# of AIMs)	# in ASW (% passed QC) *	Reference
Based on *δ *(2,076)	2,058 (100%)	This manuscript
Based on *F_ST _*(1,923)	1,907 (100%)	This manuscript
21 k random markers (21,637)	21,235 (99.97%)	This manuscript
2 k random markers (2,169)	2,130 (99.95%)	This manuscript
Tian 2000 AIMs (2,000)	1,022 (100%)	[28]
Tian 4222 AIMs (4,222)	2,125 (100%)	[28]

* AIMs in ASW HapMap data set and passing quality control for the different panels (locus call rate ≥ 95%, minor allele frequency > 0.01, and HWE *p *≥ 1.0×10^-3^).

### Application of the Admixture Panels

As an example of applying our newly developed panels, we investigated hypertension in the HUFS. The relative risk for hypertensive heart disease among African Americans was 2.80 compared to European Americans [26]. Averaged genome-wide, the individual proportion of European ancestry was 0.192 ± 0.098, 0.193 ± 0.098, and 0.264 ± 0.106 among normotensive subjects and 0.196 ± 0.119, 0.196 ± 0.119, and 0.268 ± 0.109 among hypertensive subjects, for the panels based on *δ*, *F_ST_*, and 21 k random markers, respectively. Although this result suggests that most of the differential risk in hypertension is probably not explainable by genetics, it does not preclude specific loci from significantly contributing to differential risk. Assuming the hybrid isolation model, *i.e*., a single generation of admixture with no subsequent gene flow, the estimated number of generations since the original admixture event was 7.44 ± 3.35, 7.33 ± 3.01, and 8.65 ± 5.31 for the panels based on *δ*, *F_ST_*, and 21 k random markers, respectively.

**Figure 5:**
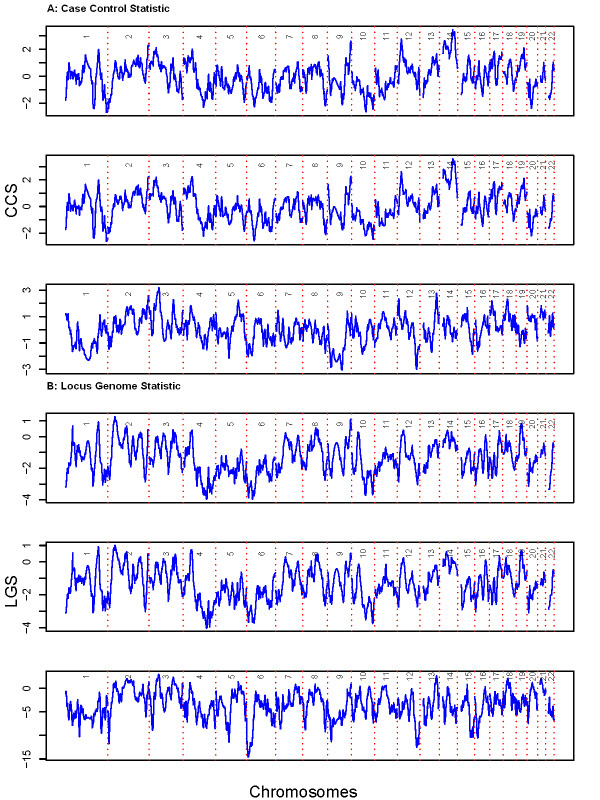
**Admixture mapping statistics scores for hypertension**. A. Locus-genome statistics for hypertension in the HUFS. Top) Panel based on *δ*. Middle) Panel based on *F*_*S*T_. Bottom) Panel based on 21 k random markers. B. Case-control statistics for hypertension in the HUFS. Top) Panel based on *δ*. Middle) Panel based on *F*_*S*T_. Bottom) Panel based on 21 k random markers.

We performed admixture mapping using both the locus-genome and case-control statistics for hypertension in the HUFS data. No marker reached genome-wide significance for hypertension case/control status using ANCESTRYMAP (Figure 5). Using a pairwise score test for markers shared between the two AIM panels, no significant difference was found between the panels (*p *= 0.8616 for the locus-genome statistics, *p *= 0.3087 for the case-control statistics). Similarly, using a *t*-test for AIMs not shared between the two panels, no significant difference was found between the panels (*p *= 0.6099 for the locus-genome statistics, *p *= 0.5607 for the case-control statistics).

## Discussion

In this study, we constructed panels of markers with variable informativeness for ancestry in admixed African Americans. We had previously genotyped our sample using the Affymetrix Genome-Wide Human SNP Array 6.0 for genome-wide association studies. Repurposing markers for admixture mapping eliminates the need for *de novo *genotyping. After linkage disequilibrium-based pruning, we constructed a set of 2,076 uncorrelated markers with large differences in allele frequencies and another set of 1,923 uncorrelated markers with large *F_ST _*values. Using these ancestry-informative markers, we estimated that the proportion of European ancestry in our sample of 1,017 unrelated African Americans from Washington, D.C. was 0.19 ± 0.11 for both panels, comparable to an estimated proportion of 0.21 ± 0.11 in a sample of 442 African Americans with multiple sclerosis and 276 controls [3]. Using a set of 21 k random markers (*i.e*., not ascertained to be informative for ancestry) in our study yielded a slightly higher estimate of admixture proportions (0.266 ± 0.108). Although it is possible to perform genome-wide admixture mapping using panels of markers not preselected to be informative for ancestry [30], our results confirm that a few thousand AIMs can be used to estimate admixture proportions as efficiently as 10-fold more random markers.

Admixed populations most commonly used in admixture mapping to date involve those formed by recent admixture between groups originating from different continents as a result of European maritime expansion during the past few hundred years [4]. The number of generations since the original admixture event based on our sample of African Americans was estimated at 7.44 ± 3.35 and 7.33 ± 3.01 generations for the panels based on *δ *and *F_ST_*, respectively. This estimate is similar to previous estimates of 6.0 ± 1.6 [3], 6.3 ± 1.1 [26], and 7 [42]. Thus, these estimates are stable across different marker panels and different samples of African Americans.

The power of admixture mapping is affected by the information content of the marker map, the sample size, and admixture proportions. We estimated that both AIM panels had an average map power of 0.73 ± 0.08, which is similar to 0.71 ± 0.09 for a previously constructed panel of 2,154 AIMs in African Americans [26]. The two panels had higher map power than the panel of 21 k random markers, which had an average map power of 0.65 ± 0.08. For the locus-genome statistic, a sample size of 500 cases provides 70% power to detect a locus conferring 1.7-fold increased risk due to ancestry [3]. Our study sample size of 509 cases and 508 controls was underpowered for loci conferring 1.5-fold or less risk due to ancestry. Although the power of admixture mapping decreases in populations with a much larger contribution from only one parental population [26], the map power is fairly constant for values of admixture proportion from 10% to 90% [3]. Our estimated values of 19% European ancestry and 81% African ancestry both fall within this range.

## Conclusions

We constructed two panels of AIMs for admixture mapping in African Americans from experimentally determined genotypes using the Affymetrix Genome-Wide Human SNP Array 6.0. We constructed the panels conditional on linkage equilibrium over 1 Mb bins. Our iterative pruning procedure was designed to avoid gaps in coverage and to eliminate background linkage disequilibrium. Given the mathematical relationship between *δ *and *F_ST_*, we recommend both panels of AIMs equally.

Developing marker panels for admixture mapping from existing genotype data derived from commercial high density SNP chips offers two major advantages. (1) No *de novo *genotyping needs to be done, thereby saving costs. (2) Markers can be filtered for various quality measures and replacement markers (to minimize gaps) can be selected at no additional cost. For our African American sample, we took advantage of preexisting HapMap genotypes for the CEU and YRI samples, but appropriate parental populations may not have already been sampled for some admixed populations. We found that the map power for sparser panels of AIMs is higher than for denser panels of 21 k random markers. Historically, the number of AIMs in an admixture panel reflected the trade-off between maximizing genomic coverage and minimizing genotyping costs. Currently, custom genotyping a panel of ~2,000 AIMs is less expensive than chip-based genome-wide genotyping. However, chip-based genome-wide genotyping is currently less expensive than custom genotyping a panel of ~20,000 random markers. Presumed parental populations are necessary to characterize AIMs. In contrast, parental populations are not needed to characterize random markers prior to estimating admixture proportions. Apart from needing many more random markers compared to AIMs, the major disadvantage of using a panel of random markers without parental populations or external reference samples is the inability to label clusters. Taken together, the ability to develop dense panels of markers from commercial chips provides a fresh opportunity to conduct admixture mapping for disease genes in admixed populations.

## Authors' contributions

GC performed the statistical analysis and drafted the manuscript. DS participated in statistical analysis and interpretation and drafted the manuscript. JZ managed the data and participated in data analysis. AD and HH conducted molecular laboratory analysis. NPG conducted molecular laboratory analysis and genotype calling. AH and MFC conceived and designed the study. YC, GMD, and MUF contributed to study coordination and reviewed the manuscript. CNR and AA conceived and designed the study, participated in data interpretation, and wrote the manuscript. All authors read and approved the final manuscript.

## Supplementary Material

Additional file 1 **Markers in the panel based on *δ***. *δ*, *F_ST_*, and SIC values for AIMS in the panel based on *δ*. Click here for file

Additional file 2 **Markers in the panel based on *F_ST_***. *δ*, *F_ST_*, and SIC values for AIMS in the panel based on *F_ST_*. Click here for file

Additional file 3 **Markers in the 2 k and 21 k random marker panels**. *δ*, *F_ST_*, and SIC values for AIMS in the panel based on 2 k and 21 k random marker panels. Click here for file

Additional file 4 **Multidimensional scaling plot.** Top four dimensions from multidimensional scaling plot showing HUFS in blue circles, CEU in red squares, and YRI in green diamonds. Click here for file

Additional file 5 **Inter-marker genetic distances (excluding centromeres)**. Average inter-marker distances in the panels based on *δ*, *F_ST_*, 2 k, and 21 k random marker. Click here for file

Additional file 6 **Distributions of *δ*, *F_ST_*, and SIC for the AIMs panels**. Genome-wide distributions of *δ*, *F_ST_*, and SIC values for AIMS. Red represents values from the panel based on *δ*, blue represents values from the panel based on *F_ST_*, and dark green represents values from the panel of 21 k random markers. Top) *δ *values. Middle) *F_ST _*values. Bottom) SIC values. Click here for file
