# Supplementary figures and images for: Development of admixture mapping panels for African Americans from commercial high-density SNP arrays
Source: BMC Genomics. 2010 Jul 5;11:417. doi: 10.1186/1471-2164-11-417 (PMC2996945; doi:10.1186/1471-2164-11-417)

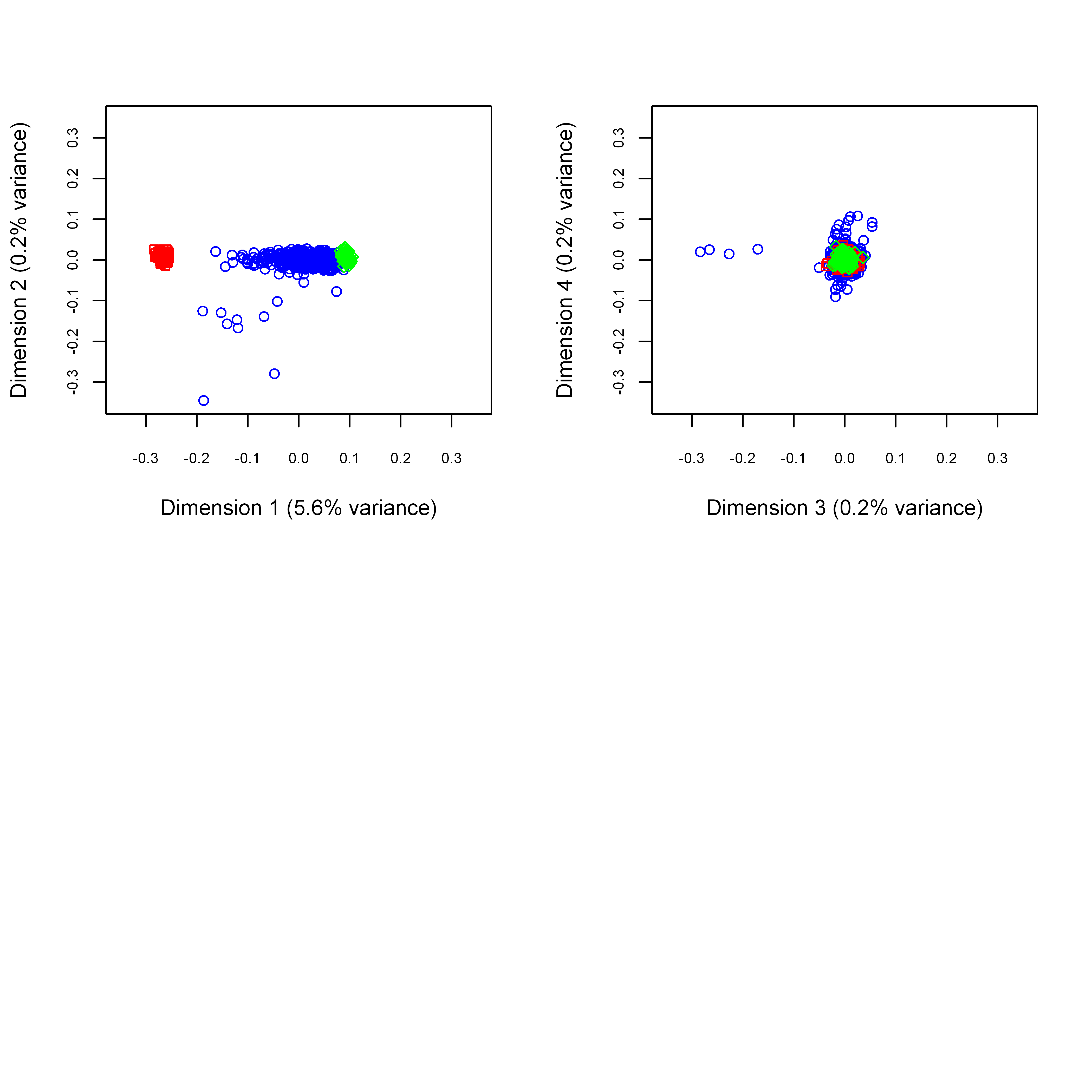

Supplement: Additional file 4 — Multidimensional scaling plot. Top four dimensions from multidimensional scaling plot showing HUFS in blue circles, CEU in red squares, and YRI in green diamonds. [file 1471-2164-11-417-S4.DOC]

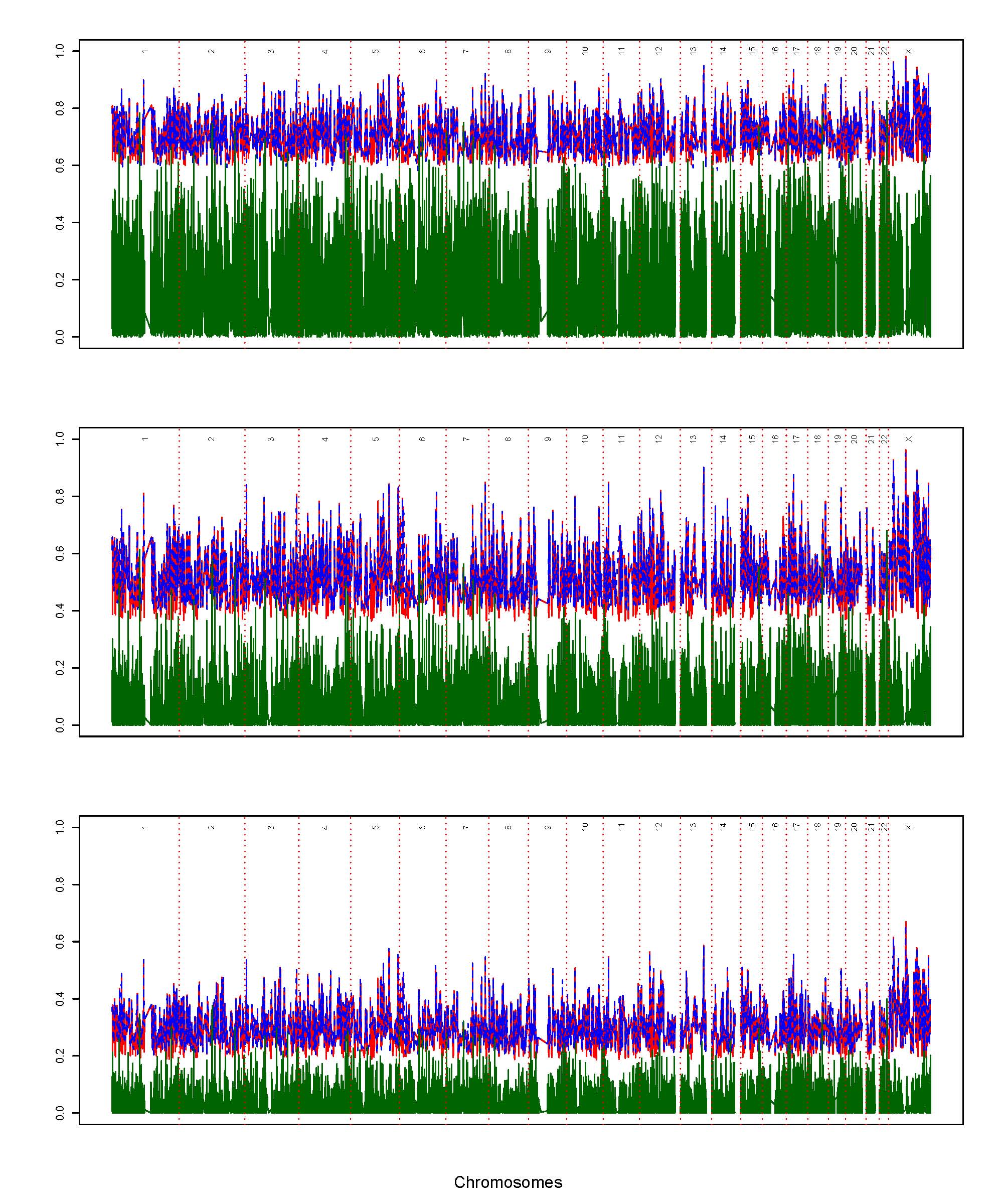

Supplement: Additional file 6 — Distributions of δ, FST, and SIC for the AIMs panels. Genome-wide distributions of δ, FST, and SIC values for AIMS. Red represents values from the panel based on δ, blue represents values from the panel based on FST, and dark green represents values from the panel of 21 k random markers. Top) δ values. Middle) FST values. Bottom) SIC values. [file 1471-2164-11-417-S6.DOC]
