# Supplementary material for: Development of admixture mapping panels for African Americans from commercial high-density SNP arrays
Source: BMC Genomics. 2010 Jul 5;11:417. doi: 10.1186/1471-2164-11-417 (PMC2996945; doi:10.1186/1471-2164-11-417)
Supplement: Additional file 5 — Inter-marker genetic distances (excluding centromeres). Average inter-marker distances in the panels based on δ, FST, 2 k, and 21 k random marker. [file 1471-2164-11-417-S5.DOC]

**Table S4.** Inter-marker genetic distances (excluding centromeres).

|  | **Panel based on *δ*** | | **Panel based on *F_ST_*** | | **Panel based on 21k random markers** | | **Panel based on 2k random markers** | |
| --- | --- | --- | --- | --- | --- | --- | --- | --- |
| **Chr** | **# of AIMs** | **Distance *** | **# of AIMs** | **Distance** | **# of**  **AIMs** | **Distance** | **# of**  **AIMs** | **Distance** |
| 1 | 167 | 1.34 (0.03, 6.39) | 150 | 1.49 (0.03, 8.88) | 1,734 | 0.13 (0, 3.28) | 171 | 1.29 (0, 11.83) |
| 2 | 184 | 1.28 (0.02, 4.84) | 177 | 1.33 (0.02, 6.23) | 1,545 | 0.15 (0, 1.85) | 155 | 1.49 (0, 7.46) |
| 3 | 139 | 1.39 (0.20, 6.38) | 132 | 1.46 (0.20, 7.79) | 1,376 | 0.14 (0, 3.17) | 137 | 1.43 (0, 8.55) |
| 4 | 139 | 1.34 (0.18, 4.72) | 131 | 1.39 (0.18, 4.24) | 1,327 | 0.14 (0, 1.98) | 143 | 1.49 (0, 7.93) |
| 5 | 115 | 1.52 (0.03, 6.30) | 106 | 1.64 (0.06, 6.50) | 1,303 | 0.14 (0, 3.41) | 133 | 1.28 (0, 6.83) |
| 6 | 107 | 1.48 (0.06, 5.30) | 95 | 1.67 (0.05, 5.30) | 1,182 | 0.14 (0, 3.64) | 146 | 1.12 (0, 7.21) |
| 7 | 112 | 1.38 (0.10, 5.19) | 104 | 1.49 (0.10, 5.19) | 1,126 | 0.14 (0, 2.24) | 98 | 1.49 (0, 8.27) |
| 8 | 115 | 1.24 (0.07, 3.80) | 107 | 1.32 (0.07, 4.45) | 1,006 | 0.14 (0, 1.60) | 97 | 1.37 (0, 9.71) |
| 9 | 82 | 1.32 (0.06, 5.51) | 76 | 1.43 (0.06, 5.51) | 1,003 | 0.12 (0, 7.48) | 91 | 1.29 (0, 8.21) |
| 10 | 105 | 1.25 (0.03, 5.16) | 98 | 1.34 (0.03, 5.16) | 1,042 | 0.12 (0, 2.02) | 107 | 1.15 (0, 8.15) |
| 11 | 92 | 1.33 (0.12, 9.47) | 84 | 1.44 (0.12, 9.47) | 965 | 0.13 (0, 1.59) | 93 | 1.32 (0, 7.00) |
| 12 | 87 | 1.45 (0.03, 4.29) | 75 | 1.69 (0.03, 5.60) | 1,072 | 0.12 (0, 1.54) | 101 | 1.16(0, 10.42) |
| 13 | 63 | 1.43 (0.04, 5.11) | 61 | 1.51 (0.04, 5.11) | 824 | 0.12 (0, 1.30) | 87 | 1.04 (0, 4.90) |
| 14 | 68 | 1.26 (0.08, 2.94) | 61 | 1.41 (0.08, 3.21) | 742 | 0.11 (0, 1.45) | 70 | 1.14 (0, 5.29) |
| 15 | 66 | 1.15 (0.07, 4.69) | 59 | 1.29 (0.07, 4.69) | 726 | 0.11 (0, 1.72) | 69 | 1.08 (0.01, 6.60) |
| 16 | 60 | 1.26 (0.05, 4.56) | 58 | 1.26 (0.02, 4.56) | 776 | 0.10 (0, 1.91) | 66 | 0.99 (0, 4.45) |
| 17 | 53 | 1.40 (0.22, 3.17) | 49 | 1.43 (0.22, 3.92) | 708 | 0.11 (0, 1.69) | 76 | 0.90 (0.01, 5.92) |
| 18 | 49 | 1.50 (0.17, 4.20) | 46 | 1.60 (0.17, 4.20) | 722 | 0.10 (0, 1.04) | 72 | 1.01 (0, 4.78) |
| 19 | 38 | 1.38 (0.29, 3.94) | 36 | 1.48 (0.18, 3.76) | 488 | 0.11 (0, 1.54) | 42 | 1.18 (0.03, 7.26) |
| 20 | 52 | 1.13 (0.29, 3.37) | 50 | 1.18 (0.27, 3.76) | 648 | 0.09 (0, 1.42) | 60 | 0.88 (0.01, 7.26) |
| 21 | 26 | 1.11 (0.21, 2.51) | 24 | 1.20 (0.34, 2.51) | 344 | 0.09 (0, 0.88) | 40 | 0.71 (0.01, 3.54) |
| 22 | 24 | 1.32 (0.04, 2.55) | 21 | 1.39 (0.04, 3.02) | 415 | 0.08 (0, 1.16) | 46 | 0.63 (0.01, 2.75) |
| X | 133 | 1.13 (0.17, 3.12) | 123 | 1.21 (0.17, 3.60) | 563 | 0.26 (0, 3.89) | 69 | 1.89 (0.01, 8.87) |

* Distances are given as mean (min, max) in cM.
